# Supplementary material for: Engagement in meaningful activities post suicide loss: A scoping review protocol
Source: PLoS One. 2024 Jan 2;19(1):e0296522. doi: 10.1371/journal.pone.0296522 (PMC10760851; doi:10.1371/journal.pone.0296522)
Supplement: S1 Appendix — (DOCX) [file pone.0296522.s001.docx]

**S1 Appendix A**

**Search strategy for MEDLINE via Ovid**

1) Suicide/ or suicide, completed/

2) (kill* adj1 (onesel* or one-sel*)).ti,ab,kf.

3) (kill* adj1 themsel*).ti,ab,kf.

4) suicid*.ti,ab,kf.

5) 1 or 2 or 3 or 4

6) exp Bereavement/

7) (bereav* or mourn* or grie* or (loss* adj4 suicid*) or (expos* adj4 suicid*)).ti,ab,kf.

8) 6 or 7

9) 5 and 8

10) limit 9 to (english language and yr="1970 -Current")
