# Supplementary material for: Engagement in meaningful activities post suicide loss: A scoping review protocol
Source: PLoS One. 2024 Jan 2;19(1):e0296522. doi: 10.1371/journal.pone.0296522 (PMC10760851; doi:10.1371/journal.pone.0296522)
Supplement: Attachment — Submitted filename: ReviewofEngagementinMeaningful Activites.docx [file pone.0296522.s003.docx]

Review of “Engagement in Meaningful Activities…A Scoping Review Protocol

The proposal proposes to review studies (published after 1969) on post-loss engagement in meaningful activities by those people who were affected by suicide. The proposal has been pre-registered on Open Science Framework Registries. The proposal follows relevant guidelines for conducting scoping reviews. I recommend its publication if the journal has a policy to publish study proposals rather than completed studies. I do have some concerns with the statement of research questions on page 6:

*1) What is the nature and extent to which the peer-reviewed, suicide loss and bereavement literature addresses engagement in meaningful activities of everyday living (i.e., occupation)?*

*2) What key facilitators and/or barriers impacting engagement in meaningful activities post-loss are described within the literature?*

The above two broadly stated research questions do not address aspects stated in the abstract (Conclusions Part):

“Experiencing a suicide loss increases the risk of negative mental health outcomes. However, engagement in meaningful activities may improve mental health outcomes and have implications for improving supports and services for those bereaved.”

It seems the proposal needs to consider also reviewing—what type of mental health concerns are reported by people who have been exposed to and affected by suicide, and suicide bereaved short and long term (as stated on page 7). The question of whether exposure, etc. increases the risk of mental health issues is not addressed in the questions. This would require reviewing studies that specifically examine those studies assessed risks associated with exposure etc. Furthermore, it would be helpful to define these terms: “exposed to” “affected by” and “bereaved short” and long term.” It would also be helpful to define “meaningful activities” – are these specifically undertaken for coping purposes (e.g., seeing a therapist, joining a self-help group, reading self-help books on bereavement)? Can continuing to engage in daily living activities (e.g., brushing one’s teeth, taking showers, eating meals) be meaningful? It is not clear whether they plan to examine if they plan on reviewing whether engaging in meaningful activities indeed improves mental health outcomes. Will they be identifying the “meaningful” activities that studies have found to improve mental health outcomes? It would be helpful to have these points clarified in the proposal.
